# Impact on Mental Health Due to COVID-19 Pandemic: Cross-Sectional Study in Portugal and Brazil

**DOI:** 10.3390/ijerph17186794

**Published:** 2020-09-17

**Authors:** Lígia Passos, Filipe Prazeres, Andreia Teixeira, Carlos Martins

**Affiliations:** 1 Department of Education and Psychology, University of Aveiro, 3810-193 Aveiro, Portugal; ligiamaria@ua.pt; 2 Faculty of Health Sciences, University of Beira interior, 6200-506 Covilhã, Portugal; 3 Family Health Unit Beira Ria, 3830-596 Gafanha da Nazaré, Portugal; 4 Centre for Health Technology and Services Research (CINTESIS), University of Porto, 4200-450 Porto, Portugal; andreiasofiat@med.up.pt (A.T.); carlosmartins20@gmail.com (C.M.); 5 Department of Community Medicine, Information and Health Decision Sciences (MEDCIDS), Faculty of Medicine, University of Porto, 4200-450 Porto, Portugal

**Keywords:** COVID-19, pandemic, mental health, well-being, depression, anxiety, Portugal, Brazil

## Abstract

Mental health effects secondary to the COVID-19 pandemic were till recently considered less important or were neglected. Portugal and Brazil are facing the pandemic in quite different ways. This study aimed to describe the mental health status of the general adult population in Portugal and Brazil during the COVID-19 pandemic and analyze the differences between the two countries. A cross-sectional quantitative study was based on an online questionnaire. Socio-demographic data were collected in addition to four validated scales: CAGE (acronym cut-annoyed-guilty-eye) Questionnaire, Satisfaction with Life Scale, Generalized Anxiety Disorder-7 and Patient Health Questionnaire-2. For each outcome, a multiple linear regression was performed. Five hundred and fifty people answered the questionnaire (435 women). The median age was 38 (Q1, Q3: 30, 47) years, 52.5% resided in Brazil and 47.5% in Portugal. The prevalence of anxiety was 71.3% (mild anxiety was present in 43.1%), the prevalence of depression was 24.7% and 23.8% of the sample had both depression and anxiety. Isolation was a significant factor for depression but not for anxiety. Well-being was below average. Mental illness was considerably higher than pre-COVID-19 levels. Portugal and Brazil will have to be prepared for future consequences of poor mental health and contribute immediate psychological support to their adult populations.

## 1. Introduction

During the current global health crisis, caused by the declaration of the coronavirus disease 2019 (COVID-19) outbreak as a pandemic on 11 March 2020 by the World Health Organization (WHO) [1], countries’ main efforts are concentrated on implementing measures to prevent, control and treat the illness caused by the severe acute respiratory syndrome coronavirus 2 (SARS-CoV-2), along with research to develop a vaccine.

The first positive case was registered in Brazil on 26 February 2020 [2] and in Portugal on 2 March 2020 [3]. In Brazil, the first death from COVID-19 occurred on March 12 [4] and 4 days later the same happened in Portugal [5].

Portugal and Brazil are facing the pandemic in quite different ways. The government of Portugal acted quickly with health precautionary measures, contingency plans and political union. On 18 March 2020, a national state of emergency was declared, suspending some citizens’ rights so that public health protection measures could be implemented. Some of the measures adopted throughout the country were: compulsory confinement at home, the establishment of sanitary fences and the closing of commercial and educational establishments, among others [6]. With good control of the number of cases across the country, after 45 days, on 2 May 2020, Portugal ended the state of emergency [7], thus returning to activities in a process of gradual de-confinement. As of 12 September 2020, Portugal had 3681 confirmed cases per 1 million of the population, and 118.1 deaths/1 M by COVID-19 [8]. In Brazil, without an official lockdown and national guidelines, prevention policies varied widely between regions, with decisions made locally by state and district governments. Most cities canceled classes, closed shops and restaurants and restricted public events. Meanwhile, other cities kept commercial and non-essential services open despite official recommendations [9]. This resulted in a lack of a uniform strategy to combat the spread of COVID-19 throughout the whole country. In early July, Brazil was considered the epicenter of the pandemic and, as of 12 September 2020, it had 20,126 confirmed cases per 1 million of the population and 613 deaths/1 M by COVID-19 [8]. Variances in social disruption policies between Portugal and Brazil may impact differently on mental health status since the length of social isolation is a risk factor for pandemic coping [10].

Mental health effects in the general population secondary to the pandemic phenomenon were till recently considered less important, or were neglected [11,12,13,14]. However, in the near future, an increase in mental health research is expected and a consequent change in health care provision and policy, as the significant impacts of COVID-19 on mental health are recognized [15].

The adverse consequences of a pandemic for mental health are complex [16]. One major consequence is likely to be increased social isolation [17], which can be characterized as the deprivation of contact, conviviality and social interactions with family, friends, neighbors and with society in general [18].

Much of what is currently known from previous viral epidemics originates from studies based on the analysis of small samples (with short periods of isolation) from SARS-CoV1, Ebola or H1N1 outbreaks, since there was no previous record of a social isolation period that covered such a large proportion of the world population and at the same time lacked a predicted isolation end date, as has occurred for the COVID-19 pandemic [19,20].

Public health actions, such as social isolation measures or prophylactic quarantine, are essential for the protection of individuals and to reduce the risk of possible contact with SARS-CoV-2, but at the same time, these individuals experience a high burden of mental health conditions [21,22]. As time lived in isolation increases, the greater are the chances of triggering psychological diseases [23]. Depressed mood, irritability, anxiety, high stress levels and insomnia are a few of the common examples of specific mental health outcomes associated with isolation [20,22]. In the long term, the impact of social isolation can also contribute to the abuse of alcohol [24] and/or other substances, and also to family violence [25].

Existing research recognizes the critical role played by the COVID-19 pandemic in mental health. A recently published literature review that included 15 articles regarding the mental health outcomes associated with the COVID-19 pandemic in non-clinical populations found that 7% to 53.8% of Chinese people experienced mostly anxiety, depression and stress during the COVID-19 outbreak [16]. Other studies with different populations also showed high levels of psychological distress (72.0% in Spain) [26] and the deterioration of mental health status since the start of the pandemic in Hong Kong [27]. In Italy, prevalence of depression and anxiety was found to be 24.7% and 23.2% [28]. In North America, a prevalence of 44.1% for depression and 47.2% for anxiety was observed in Canada [29], and in the USA, 24.4% and 28.2%, respectively [30].

The analysis of the consequences of social distancing during the current pandemic crisis is now even more important, as several European countries (e.g., Spain, Portugal, the United Kingdom, Ireland, France, Belgium and Germany) are returning to more restrictive measures due to the increase in new cases of infection by SARS-CoV-2 and the threat of a COVID-19 second wave [31,32].

The present study aimed to describe the mental health status of the general adult population in Portugal and Brazil during the COVID-19 pandemic and analyze the differences between the two countries of the impact on the emotional well-being of the decrease in interpersonal contact due to COVID-19.

## 2. Materials and Methods

### 2.1. Study Design

This is a cross-sectional quantitative study based on an online questionnaire conducted from 27 May to 8 July 2020, among adults from the general population living in Portugal or Brazil.

### 2.2. Setting and Participants

For inclusion in the study, participants should be residents in Portugal or Brazil, be over 18 years old, give their informed consent and agree to participate in the study. The questionnaire was built in the Google Forms platform and the questionnaire web link was sent by e-mail to the researchers’ contact network, and through community groups in social networks, thus generating a snowball sample, where invited respondents shared the online questionnaire with their contacts.

### 2.3. Measurements

Socio-demographic and other factors: variables assessed included age, gender, country of residence (Portugal or Brazil), marital status, educational level, employment status, social isolation self-label, duration of social isolation, living arrangements during social isolation, diagnosis of COVID-19, alcohol consumption and alcohol addiction measured by the presence of two or more positive answers to the four-item CAGE (acronym cut-annoyed-guilty-eye) Questionnaire [33,34] translated and validated for the Portuguese language [35].

Satisfaction With Life Scale (SWLS): a global cognitive measure of satisfaction with one’s life [36,37]. It consists of five items rated on a five-point Likert scale, ranging from 1: “strongly disagree” to 5: “strongly agree”. This scale was translated and validated for the Portuguese language [38]. A total score is obtained by the sum of the five items (range from 5 to 25 points). Cronbach’s alpha for this scale was 0.87 as reported by Diener et al. (1985) [37] and 0.88 for the current sample.

Generalized Anxiety Disorder-7 (GAD-7): a brief self-report scale to identify probable cases of generalized anxiety disorder and assess its severity in both the primary care setting and the general population [39,40,41]. The seven items of this instrument are scored on a four-point Likert scale where 0: “not at all”; 1: “several days”; 2: “more than half the days”; and 3: “nearly every day”. A total score is obtained by the sum of the seven items (range from 0 to 21 points). The cut-off points for classifying the severity of anxiety are: 0–4 = none/normal, 5–9 = mild, 10–14 = moderate and 15–21 = severe. GAD-7 was validated for the Portuguese language by Sousa et al. (2015) [42]. In the present study, GAD-7 was found to have excellent internal consistency (Cronbach’s alpha of 0.90). For the purpose of the current study, a total score of five points or above was used to indicate the possible presence of anxiety [42].

Patient Health Questionnaire-2 (PHQ-2): a two-item depression screener. It includes the first two items of the PHQ-9 [43,44] and evaluates the frequency of depressed mood and anhedonia. Some authors consider PHQ-2 more explanatory than using all the PHQ-9 questions [45]. The two items of this instrument are scored on a four-point Likert scale where 0: “not at all”; 1: “several days”; 2: “more than half the days”; and 3: “nearly every day”. The sum score ranges from 0 to 6 points. A total score of 3 or above indicates that major depressive disorder is likely [46,47]. In the present study, PHQ-2 was found to have good internal consistency (Cronbach’s alpha of 0.83).

### 2.4. Statistical Analysis

Data analysis was performed using SPSS 26.0 version statistical software (IBM, Armonk, NY, USA) and Jamovi (Version 1.2) (Computer Software, Sydney, Australia). Categorical variables were described using absolute and relative frequencies, *n*(%) or (*n*; %). The prevalences are presented with the respective 95% confidence intervals. Continuous variables not normally distributed were described by the median and the interquartile interval, Mdn (Q_1_, Q_3_). The normality of continuous variables was assessed by observation of Q–Q plots.

The comparison of continuous variables between Portugal and Brazil was made by the Mann–Whitney test since the variables were not normally distributed. The comparison of categorical variables was made by the chi-squared test.

For each outcome—satisfaction with life (SWLS), anxiety (GAD-7) and depression (PHQ-2)—a separated multiple linear regression was performed. To decide which independent variables to include in each multiple regression, simple linear regressions were performed with each variable in the dataset, including socio-demographics, variables related to COVID-19 and emotional variables, were obtained from questionnaires: satisfaction with life (SLWS), anxiety (GAD-7) and depression (PHQ-2). All variables that correlated with the outcomes at *p* ≤ 0.20 in a simple regression were included in the multiple linear regressions [48]. Only the significant variables were maintained in the final multiple models. The results of linear regressions were presented by the coefficient values (β) and the respective *p*-value. To evaluate the model, the determination coefficient (r^2^) was presented. Assumptions of the linear regression models were verified as follows: (1) visual analysis of histograms to assess the normality of residuals and (2) plotting residuals versus the fitted predictive values for checking homoscedasticity. Values of *p* ≤ 0.05 were considered significant.

### 2.5. Sample Size

The sum of the Portuguese and Brazilian populations over 18 years of age are approximately 164,058,140 [49,50]. The minimum sample size (*n* = 385) was calculated for proportions and considering the most conservative scenario (a proportion of 50%), a population of 164,058,140 individuals, a level of confidence of 95% and an error margin of 5%.

### 2.6. Ethics

The present study followed the Declaration of Helsinki ethical standards and was approved by the Ethics Committee of the University of Beira Interior (CE-UBI-Pj-2020-041). Electronic consent was obtained from all participants. Responses were anonymous.

## 3. Results

The questionnaire was answered by 550 participants, 289 (52.5%) residing in Brazil and 261 (47.5%) in Portugal. This sample size corresponds to a margin of error of 4.18% (in the same conditions of the sample size calculator). All participants fully completed SWLS, GAD-7 and PHQ-2 instruments. No participants were excluded from the analysis. The characteristics of the participants are summarized in Table 1. Most of them were female (435; 79.5%). The median age was 38 (Q1, Q3: 30, 47) years. Regarding marital status, 290 (52.8%) were married or cohabiting. The level of education was high, with 51.3% (*n* = 282) being postgraduates, masters or PhDs and 61.6% (*n* = 335) declared being employed. Notice that 88.2% of participants (*n* = 485) were in social isolation, with a median duration of 70 (Q1, Q3: 60, 90) days, and of these 485 participants, 81.4% (*n* = 395) had experienced more than 51 days in social isolation. Most participants (383; 69.6%) lived with their families during this period. Only 12.2% of participants (*n* = 67) were tested for COVID-19 and only 1.3% (*n* = 7) tested positive. More than half (310; 56.4%) reported consuming alcoholic beverages. Alcohol addiction (two or more points on CAGE) was present in 10.3% (*n* = 31) of the respondents, without a statistical difference between the residents of the two countries (chi-squared test; *p* = 0.995).

A chi-squared test of independence was performed to examine the relation between the country of residence and isolation. Residents in Brazil were more likely than residents in Portugal to isolate (*p* = 0.003); and the length of the isolation period was more likely to be longer than 51 days in Brazil (*p* < 0.001).

Respondents scored slightly below average in life satisfaction (SWLS), with a median score of 18 (Q1, Q3: 14, 21) points and there were no significant differences between residents of Portugal and Brazil (*p* = 0.292; Table 2). Considering GAD-7, the median score was 6 (Q1, Q3: 4, 11) points, also without significant differences between residents of Portugal and Brazil (*p* = 0.113; Table 2). The prevalence rate of anxiety was 71.3% (95% CI, 67.5–75.1) (mild anxiety was present in 43.1% (95% CI, 39.0–47.2), moderate anxiety in 17.6% (95% CI, 14.5–21.1) and severe anxiety in 10.5% (95% CI, 8.0–13.1) of the sample). The median PHQ-2 score was 2 (Q1, Q3: 0, 2) points and residents of Brazil had a slight but significantly higher median score than Portuguese ones (2 vs. 1, *p* = 0.040; Table 2). The prevalence rate of depression was 24.7% (95% CI, 21.1–28.3) and 23.8% (95% CI, 20.3–27.4) had both depression and anxiety. No differences were found in the prevalence of having both anxiety and depression between the Portuguese and Brazilian subgroups (*p* = 0.059 and *p* = 0.273, respectively; Table 2).

Gender, educational level, professional status, co-living status and depression (PHQ-2 score) were found to be significant factors for life satisfaction (SWLS) in multiple linear regression (r^2^ = 0.211; Table 3). Women’s life satisfaction scores were higher by an average of 1.08 in comparison to men (*p* = 0.027). Higher levels of education were significantly associated with increased levels of life satisfaction scores (β = 1.81, *p* = 0.006). Students’ life satisfaction scores were higher by an average of 1.56 in comparison to employees (*p* = 0.002). Those who lived with family members or with a partner in the period of social isolation were significantly associated with increased levels of life satisfaction (β = 1.59, *p* = 0.023 and β = 2.53, *p* = 0.001, respectively). Higher levels of depression (PHQ-2) were significantly associated with a reduction of life satisfaction levels (β = −1.26, *p* < 0.001).

Gender and depression (PHQ-2 score) were found to be significant factors for anxiety (GAD-7) in multiple linear regression (r^2^ = 0.462; Table 4). Women’s anxiety levels were higher by an average of 0.88 in comparison to men (*p* = 0.020). Higher levels of depression (PHQ-2) were significantly associated with an increase in anxiety levels (β = 2.03, *p* < 0.001).

Social isolation, life satisfaction (SWLS) and anxiety (GAD-7) were found to be significant factors for depression (PHQ-2) in multiple linear regression (r^2^ = 0.519; Table 5).

Being in social isolation was significantly associated with an increase in depression levels (β = 0.33, *p* = 0.026). Higher levels of life satisfaction (SWLS) were significantly associated with a reduction of depression levels (β = −0.07, *p* < 0.001). Higher levels of anxiety (GAD-7) were significantly associated with an increase in depression levels (β = 0.20, *p* < 0.001).

All levels of anxiety had a significant association with the PHQ-2 scale, in comparison with the group without/normal anxiety levels. Those with severe anxiety had a depression level that was on average higher by 3.14 (*p* < 0.001).

## 4. Discussion

To the extent of the authors’ knowledge, the present study is the first to analyze the mental health status of the general adult population in Portugal and Brazil during the COVID-19 pandemic. It has been previously expressed that mental health conditions are going to be the great pandemic of this century [51] and, to some extent, the results of the current study corroborate this statement.

In the present study, the prevalence of anxiety was 71.3% (mild anxiety was present in 43.1%), the prevalence of depression was 24.7% and 23.8% of the sample had both depression and anxiety. The observed frequency of mental illness was considerably higher than pre-COVID-19 levels, as expected from the results of previous studies that suggested a connection between a public health crisis and mental health conditions [52,53]. Even before the COVID-19 outbreak, Brazil had the highest prevalence of anxiety among all countries in the world, with 9.3% of the population having some type of anxiety disorder. At the same time, the prevalence of anxiety in Portugal was 4.9%. Regarding depressive disorders, the prevalence was similar in both countries (5.7% vs. 5.8% for Portugal and Brazil, respectively) [54].

Even though the studies were done during the initial stage of the COVID-19 outbreak [16] and used different scales or populations so no direct comparison between studies is possible, the present study showed a similarly high prevalence of mental health conditions (e.g., the prevalence rate of depression was 50.7% and that of generalized anxiety was 44.7% in a multicenter study involving around one and a half thousand Chinese medical workers [55]). It can thus be suggested that the COVID-19 pandemic has significantly affected the mental health of the general adult population in Portugal and Brazil, with an increased risk of future challenges of impairment, alcohol or drug coping, negative religious coping, hopelessness and suicidal ideation, as was the case in other samples with high levels of anxiety related to COVID-19 [56].

Female gender was associated with higher levels of psychological distress in the time of COVID-19 [16]. In the present study, women were associated with higher rates of anxiety but not depression. This finding is contrary to previous studies which have suggested that women are at higher risk of anxiety and depression [57,58]. This inconsistency may be due to the effect of the current public health crisis on the use of coping strategies, like positive reframing [59]. Although females showed higher anxiety in comparison to males, this did not affect their self-rated life satisfaction. This could be an important issue for future research.

In Portugal, the social confinement lasted about 51 days for many people [21] (and much more in Brazil, although with state dissimilarities), despite that, the individuals that were isolated had higher depression levels. This result suggests that social isolation may be a risk factor for depression, in agreement with the results obtained by previously published research [60,61], and this may be even more evident when a quarantine is imposed [20] or if loneliness is present [62].

One interesting finding of this study was that during the ongoing COVID-19 pandemic, anxiety and depressive disorders continue to be particularly comorbid [63], with higher levels of depression being significantly associated with increasing levels of anxiety and vice versa. Prior studies have noted that almost half of depressive individuals (45.7%) have had an anxiety disorder during their life [64] and that 42% of individuals with anxiety have had at least one episode of depression in their life [65]. An implication of the current study’s results is the possibility that individuals experiencing the COVID-19 pandemic are at higher risk of developing more severe symptoms and poor treatment response for depression/anxiety [66].

Regarding the impact of the COVID-19 pandemic on well-being, respondents scored slightly below average on the life satisfaction scale (SWLS), without significant differences between residents of Portugal and Brazil; these scale results may be due to the presence of a meaningful problem in only one area of their lives, or more likely, in the current pandemic crisis, to the presence of problems in several areas of the respondents’ lives [67], from multiple potential stressors, such as respondents’ worries for their finances, health or those close to them, social isolation and loneliness, loss of pre-COVID routines and contact with former sources of positive reinforcement [68], which in turn may increase the risk of depression. Some of these areas were confirmed by the significant factors for life satisfaction found in multiple linear regression: professional status, educational level, co-living status and depression. In the present sample, a considerable effect on the well-being of working individuals (that students would not experience) may be due to the presence of feelings of anxiousness regarding future work and finances and this may be even more prominent in individuals with lower educational levels whose jobs are not suitable for teleworking (or remote working) during social distancing measures [69]. An implication of this is the possibility that workers with low educational levels may benefit from being well informed about their sick pay and benefits rights during the current pandemic.

### Limitations

Some limitations must be considered when interpreting the results of the present study. One limitation concerns convenience sampling and, although it was carried out in two countries, it still does not allow for the generalization of results since respondents shared similar demographic characteristics. The lack of a diverse sample limits the ability to explore how some demographic characteristics (e.g., socioeconomic status) may affect mental health during the pandemic. Portugal and Brazil faced the pandemic in different ways (e.g., social distancing measures), and distinct societal and economic characteristics between the two can still have an impact on the mental health of each population and may increase poverty and inequalities between the two countries. Some risk factors of poor mental health (or its protective factors) were not collected and therefore their role in determining the results of the present study cannot be calculated. Further work is needed to evaluate the social, environmental and economic determinants of mental health in the COVID-19 pandemic. The role of uncertainty stress on the development of mental ill-health should also be studied.

Another limitation is the fact that no information regarding the previous mental health of the participants was collected. Thus, it is not possible to analyze the extent to which the COVID-19 pandemic contributed to an expected worsening of depressive and anxiety symptoms. Future longitudinal studies could contribute to a better understanding of the late effects of social isolation on the mental health of adults.

## 5. Conclusions

Evidence suggests that the presence of mental illness was considerably higher than pre-COVID-19 levels, both in Portugal and Brazil. The prevalence of anxiety was 71.3% (mild anxiety was present in 43.1%), the prevalence of depression was 24.7% and 23.8% of the sample had both depression and anxiety. Consequently, well-being was below average. Portugal and Brazil will have to be prepared for future consequences of poor mental health and contribute immediate psychological support to their adult population.

The development and improvement of mental health public policies must be an essential part of governments’ response to the COVID-19 pandemic, with a commitment to support and care for affected individuals. The first step should be to campaign to raise public awareness about mental illnesses so not only those with issues seek early help but also those who are at increased risk (e.g., females and those in social isolation). Mental health services must be expanded and widely funded, as part of the universal health coverage, and health professionals should be knowledgeable regarding the risk factors and protective factors of mental disorders and be able to provide in-person or virtual counseling or therapy.

To improve well-being during a crisis like the COVID-19 pandemic, there is the need to maintain social connections, decrease isolation and care for the mental health of individuals by the use of, for example, phone calls or video chats with friends and loved ones.

Governments should also protect employees from being fired for being in quarantine or social isolation.

## Figures and Tables

**Table 1:** Socio-demographic data (*n* = 550).

Characteristics	
**Gender**, (*n* = 547), *n* (%)	
Female	435 (79.5)
Male	112 (20.5)
**Age** (years), Mdn (Q_1_, Q_3_)	38 (30, 47)
Youth (<25 years), *n* (%)	88 (16)
Adults (25–64 years), *n* (%)	436 (79.3)
Seniors (≥65 years), *n* (%)	26 (4.7)
**Marital Status**, (*n* = 549), *n* (%)	
Single/divorced/widowed	259 (47.2)
Married/cohabiting	290 (52.8)
**Educational Level**, *n* (%)	
High school and below	65 (11.8)
University degree	203 (36.9)
Postgraduate/Master’s/PhD	282 (51.3)
**Country of Residence**, *n* (%)	
Brazil	289 (52.5)
Portugal	261 (47.5)
**Professional Status**, (*n* = 544), *n* (%)	
Employed	335 (61.6)
Unemployed/retired	85 (15.6)
Student	124 (22.8)
**Is or Has been in Social Isolation**, *n* (%)	
Yes	485 (88.2)
No	65 (11.8)
**Duration of Social Isolation** (days), (*n* = 485), Mdn (Q_1_, Q_3_)	70 (60, 90)
≤51 days	90 (18.6)
>51 days	395 (81.4)
**Co-Living during Social Isolation**, *n* (%)	
Alone	52 (9.5)
Family	383 (69.6)
Partner	104 (18.9)
Friends	11 (2.0)
**Diagnosis of COVID-19**, *n* (%)	
Not tested	483 (87.8)
Negative	60 (10.9)
Positive with symptoms	2 (0.4)
Positive without symptoms	5 (0.9)
**Alcohol Consumption**, *n* (%)	
Yes	310 (56.4)
No	240 (43.6)
**CAGE**, (*n* = 302), *n* (%)	
No alcohol addiction (<2 points)	271 (89.7)
Alcohol addiction (≥2 points)	31 (10.3)

**Table 2:** Comparison of the mental health status between the two countries of residence (Portugal vs. Brazil; *n* = 550).

Mental Health Status Variables	Total	Portugal	Brazil	*p*-Value
	(*n* = 550)	(*n* = 261)	(*n* = 289)	
**Life satisfaction (SWLS)** (score 5–25), Mdn (Q_1_, Q_3_)	18 (14, 21)	19 (14, 21)	18 (13, 21)	0.292 ^a^
**GAD-7** (score 0–21), Mdn (Q_1_, Q_3_)	6 (4, 11)	6 (4, 10)	7 (4, 11)	0.113 ^a^
**Anxiety**, % (*n*)				0.059 ^b^
Without anxiety (score 0–4)	28.7 (158)	32.6 (85)	25.3 (73)	
With anxiety (score 5–21)	71.3 (392)	67.4 (176)	74.7 (216)	
**PHQ-2** (score 0–6), Mdn (Q_1_, Q_3_)	2 (0, 2)	1 (0, 2)	2 (1, 3)	0.040 ^a,^*
**Depression**, % (*n*)				0.273 ^b^
Without depression (score 0–2)	75.3 (414)	77.4 (202)	73.4 (212)	
With depression (score 3–6)	24.7 (136)	22.6 (59)	26.6 (77)	
**With depression and anxiety**, % (*n*)	23.8 (131)	21.5 (56)	26.0 (75)	0.216 ^b^

^a^: Mann–Whitney test. ^b^: Chi-squared test. *: statistically significant at 5%.

**Table 3:** Regression coefficients for Satisfaction with Life Scale (SWLS) as an outcome with socio-demographic and emotional variables as predictors, from univariate multiple linear regressions.

Socio-Demographic and Emotional Characteristics	Initial Model (r^2^ = 0.241)		Final Model (r^2^ = 0.211)	
	SWLS	*p*-Value	SWLS	*p*-Value
**Gender**				
Male	Reference		Reference	
Female	1.41 (0.44, 2.38)	0.004	1.08 (0.13, 2.04)	0.027
**Marital Status**				
Single/divorced/widowed	Reference			
Married/cohabiting	0.81 (−0.18, 1.81)	0.108	-	-
**Age**				
Youth	Reference			
Adults	−1.15 (−2.90, 0.60)	0.196	-	-
Seniors	0.97 (−1.63, 3.56)	0.465	-	-
**Educational Level**				
High school and below	Reference		Reference	
University degree	1.25 (−0.08, 2.57)	0.065	1.15 (−0.16, 2.46)	0.085
Postgraduate/master’s/PhD	1.89 (0.52, 3.26)	0.007	1.81 (0.51, 3.11)	0.006
**Professional Status**				
Employed	Reference		Reference	
Unemployed/retired	−0.94 (−2.10, 0.22)	0.111	−0.78 (−1.88, 0.33)	0.167
Student	1.67 (0.10, 3.23)	0.037	1.56 (0.56, 2.57)	0.002
**Country of Residence**				
Portugal	Reference			
Brazil	0.08 (−0.87, 1.04)	0.865	-	-
**Is or Has been in Social Isolation**				
No	Reference			
Yes	−0.75 (−1.97, 0.47)	0.230	-	-
**Co-living during Social Isolation**				
Alone	Reference		Reference	
Family	1.51 (0.00, 3.02)	0.049	1.59 (0.22, 2.95)	0.023
Partner	2.37 (0.64, 4.10)	0.007	2.53 (0.99, 4.08)	0.001
Friends	1.65 (−1.35, 4.65)	0.281	1.52 (−1.49, 4.53)	0.322
**Diagnosis of COVID-19**				
Not tested	Reference			
Negative	1.01 (−0.24, 2.26)	0.114	-	-
Positive with symptoms	3.72 (−0.77, 8.21)	0.104	-	-
Positive without symptoms	−1.84 (−8.16, 4.48)	0.568	-	-
**Alcohol Consumption**				
No	Reference			
Yes	0.76 (−0.05, 1.57)	0.067	-	-
**Anxiety (GAD-7)**	−0.11 (−0.22, −0.01)	0.040	-	-
**Depression (PHQ-2)**	−0.97 (−1.31, −0.64)	<0.001	−1.26 (−1.50, −1.01)	<0.001

**Table 4:** Regression coefficients for Generalized Anxiety Disorder-7 (GAD-7) as an outcome with socio-demographic and emotional variables as predictors, from univariate multiple linear regressions.

Socio-Demographic and Emotional Characteristics	Initial Model (r^2^ = 0.464)		Final Model (r^2^ = 0.462)	
	**GAD-7**	***p*-Value**	**GAD-7**	***p*-Value**
**Gender**				
Male	Reference		Reference	
Female	0.92 (0.18, 1.66)	0.016	0.88 (0.14, 1.62)	0.020
**Country of Residence**				
Portugal	Reference			
Brazil	0.16 (−0.44, 0.76)	0.605	-	-
**Life Satisfaction (SWLS)**	−0.04 (−0.10, 0.03)	0.262	-	-
**Depression (PHQ-2)**	1.98 (1.78, 2.19)	<0.001	2.03 (1.85, 2.22)	<0.001

**Table 5:** Regression coefficients for Patient Health Questionnaire-2 (PHQ-2) as an outcome with socio-demographic and emotional variables as predictors, from univariate multiple linear regressions.

Socio-Demographic and Emotional Characteristics	Initial Model (r^2^ = 0.523)		Final Model (r^2^ = 0.519)	
	PHQ-2	*p*-Value	PHQ-2	*p*-Value
**Marital Status**				
Single/divorced/widowed	Reference			
Married/cohabiting	−0.31 (−0.55, −0.07)	0.011	-	-
**Age**				
Youth	Reference			
Adults	−0.15 (−0.48, 0.17)	0.361	-	-
Seniors	−0.42 (−0.95, 0.11)	0.124	-	-
**Country of Residence**				
Portugal	Reference			
Brazil	0.17 (−0.04, 0.39)	0.116	-	-
**Is or Has been in Social Isolation**				
No	Reference		Reference	
Yes	0.29 (−0.01, 0.58)	0.057	0.33 (0.04; 0.62)	0.026
**Co-Living during Social Isolation**				
Alone	Reference			
Family	−0.05 (−0.41, 0.32)	0.795	-	-
Partner	0.06 (−0.36, 0.47)	0.795	-	-
Friends	−0.04 (−0.77, 0.70)	0.925	-	-
**Life Satisfaction (SWLS)**	−0.07 (−0.09, −0.05)	<0.001	−0.07 (−0.08, −0.05)	<0.001
**Anxiety (GAD-7)**	0.20 (0.18, 0.22)	<0.001	0.20 (0.18, 0.22)	<0.001
